# Traumatic Brain Injury in Pregnancy: A Single Level I Trauma Center Experience

**DOI:** 10.7759/cureus.93709

**Published:** 2025-10-02

**Authors:** Hector Fuentes Plata, Nisha K Dabhi, Shankar P Gopinath, Spyridoula Tsetsou

**Affiliations:** 1 Department of Neurosurgery, Baylor College of Medicine, Houston, USA; 2 Department of Neurology, Baylor College of Medicine, Houston, USA

**Keywords:** complications in pregnancy, critical care in pregnancy, neurosurgical interventions, trauma in pregnancy, traumatic brain injury

## Abstract

Traumatic brain injury (TBI) is less common in pregnancy than in the general population; however, it poses significant challenges in diagnosis and management. These include the potential teratogenicity of medications, radiation exposure from imaging modalities, physiological changes associated with pregnancy, and obstetric complications that may arise. Although there are general guidelines for trauma in the obstetric patient, they are not specific to TBI, and most recommendations are based on low-level evidence or institutional experiences.

We conducted a retrospective review of the hospital trauma registry over a 10-year period. We included all the patients admitted to our neurosurgical intensive care unit for TBI and confirmed pregnancy. The data was obtained through a chart review.

Among 2,027 TBI admissions, four patients were pregnant. Of these four patients, two underwent external ventricular drain placement; one required a decompressive craniectomy. Two patients delivered viable infants via cesarean section. Two patients were discharged home, and one to a personal care home. One patient was pronounced brain dead.

Our data confirmed the rarity of pregnancy in TBI. Even though this represents an infrequent clinical scenario, the complications and treatment can be challenging for clinicians. Therefore, pooled data from other level I trauma centers should be studied to formulate appropriate therapeutic directions, and the inclusion of this population in medical societies’ guidelines is necessary.

## Introduction

Traumatic brain injury (TBI) in obstetric patients is uncommon compared to the general population; nevertheless, mortality ranges from 30% to 50%, and around 40% experience permanent disability [1]. The reporting incidence in the United States is as low as ≈0.04%, based on a study that included 9 million deliveries over a 10-year period [2]. It occurs more frequently in the third trimester, with motor vehicle accidents (MVAs) and domestic partner violence as the leading causes [3,4].

When it comes to management, ionizing radiation, teratogenicity of medications, maternal physiology, and other pregnancy-related complications associated with trauma, such as preterm labor and placental abruption, all demand special considerations [2,5-8]. Secondary to physiological changes, there is a notable decrease in systemic vascular resistance, which leads to adjustments in myocardial contractility and renal homeostatic mechanisms, resulting in capillary engorgement and tissue edema. These factors may complicate procedures such as intubation [1]. This airway management challenge is also exacerbated by a higher risk of complications, derived from weight gain, decreased functional residual capacity, and increased oxygen demand [5]. Additionally, the increased circulating blood volume and dilutional anemia may delay the recognition of hemorrhagic shock, even when the patient's clinical stability is apparent, as signs of shock often emerge late after a large amount of blood has been lost [1,5]. Furthermore, there is an increase in clotting factors such as I, VIII, IX, and X, which leads to a prothrombotic state that is protective against peripartum hemorrhage; nevertheless, it increases the risk of disseminated intravascular coagulation and deep venous thrombosis in the trauma setting [1].

Although there are guidelines for trauma in pregnant patients, namely from the Society of Obstetricians and Gynaecologists of Canada (SOGC) and the Eastern Association for the Surgery of Trauma (EAST), they are not specific to TBI, and many of the available recommendations lack Level I evidence [1,3]. The most recent guidelines from the American College of Surgeons provide comprehensive recommendations for the management of TBI; however, they do not include pregnancy considerations [9]. Low described incidence and the fact that pregnancy is a significant exclusion criterion in most clinical trials could potentially explain this omission. Consequently, recommendations based on case reports, case series, and institutional experiences have been proposed, which may differ significantly [3,6,10]. This highlights the importance of available case studies to contribute to the development of this relatively unexplored area. This study aims to provide insights into presentation, management, and considerations for these patients in a Level I Trauma Center over a 10-year period. We conducted a retrospective review of the hospital trauma registry from January 1, 2015, to March 31, 2025. We included all patients admitted with confirmed pregnancy (urine test) and diagnosis of any severity of TBI that required hospitalization in the neurosurgical intensive care unit (NICU) of our Level I Trauma Center. Data were retrieved from the hospital trauma registry and electronic medical records. The collected data included demographics, mechanism of injury, Glasgow Coma Scale (GCS) score, imaging findings, surgical interventions, gestational age at presentation, obstetric interventions, and both maternal and fetal outcomes.

This protocol was reviewed and approved by the Institutional Review Board for Baylor College of Medicine and Affiliated Hospitals (No. H-57153). No subject contact was made, and the requirement for informed consent was waived in accordance with local protocol.

## Case presentation

During the included study period, 2,027 patients with TBI were admitted to our trauma hospital. Among them, we identified four pregnant patients, accounting for nearly 0.2% of all TBI admissions in our NICU. Three of the four patients suffered severe injuries. EVD placement was required in two patients, and one underwent decompressive craniectomy. Two of them underwent cesarean section and delivered viable infants. Two patients were discharged home, and one was sent to a personal care home. One patient was pronounced brain dead. The demographic, clinical characteristics, interventions, and outcomes of these patients are summarized in Table 1.

**Table 1:** Demographic, clinical characteristics, interventions, and outcomes of patients EVD: external ventricular drain, PbtO₂: brain tissue oxygen monitoring, GCS: Glasgow Coma Scale, GOSE: Glasgow Outcome Scale-Extended, MVA: motor vehicle accident

Patient	1	2	3	4
Age	25	35	26	20
Mechanism of trauma	MVA	MVA	MVA	Gunshot wound to the head
CT findings	Traumatic subarachnoid hemorrhage; intraventricular hemorrhage; effacement of basal cisterns; diffuse cerebral edema	Sphenoid fracture; intraventricular hemorrhage; trace bifrontal subarachnoid hemorrhage; temporal lobe contusion	Right hemispheric subdural hemorrhage; multifocal subarachnoid hemorrhage; parenchymal hemorrhage within the pons; transtentorial and tonsillar herniation	Small left parietal contusion
GCS on arrival	6T	4T	4T	15
Mechanical ventilation	Intubated on arrival for airway protection	Intubated on arrival for airway protection	Intubated on arrival for airway protection	Not required
Neurosurgical procedures	EVD and PbtO_2_ monitor	EVD and PbtO_2_ monitor	Right fronto-parieto-temporal craniectomy for evacuation of subdural hematoma	None
Need for intracranial hypertension treatment	Catheters removed on day 3	Catheters removed on day 4	Decompressive craniectomy; mannitol	Not required
Pregnancy age	35 weeks 0 days	20 weeks 2 days	14 weeks 5 days	16 weeks 1 day
Emergent C-section	Emergent low transverse cesarean section	No. Repeat low transverse cesarean delivery at 38 weeks	None	None
Viable infant, age	Viable male infant, 35 weeks 0 days; Apgar: 2/4/5	Viable male infant, 38 weeks 3 days; Apgar: 1/6/8	Previable pregnancy	Previable pregnancy
Pregnancy-related complications	None	None	Previable pregnancy	Previable pregnancy
Outcome at discharge	Discharged to a personal care home. Non-ambulatory. Tolerating bolus tube feeds. Following commands	Discharged home. Following commands. Required long-term rehabilitation	Brain death	Discharged home. At neurologic baseline. Follow-up in the neurosurgery clinic
GOSE at discharge	3	3	1	8

Patient 1

**Figure 1:**
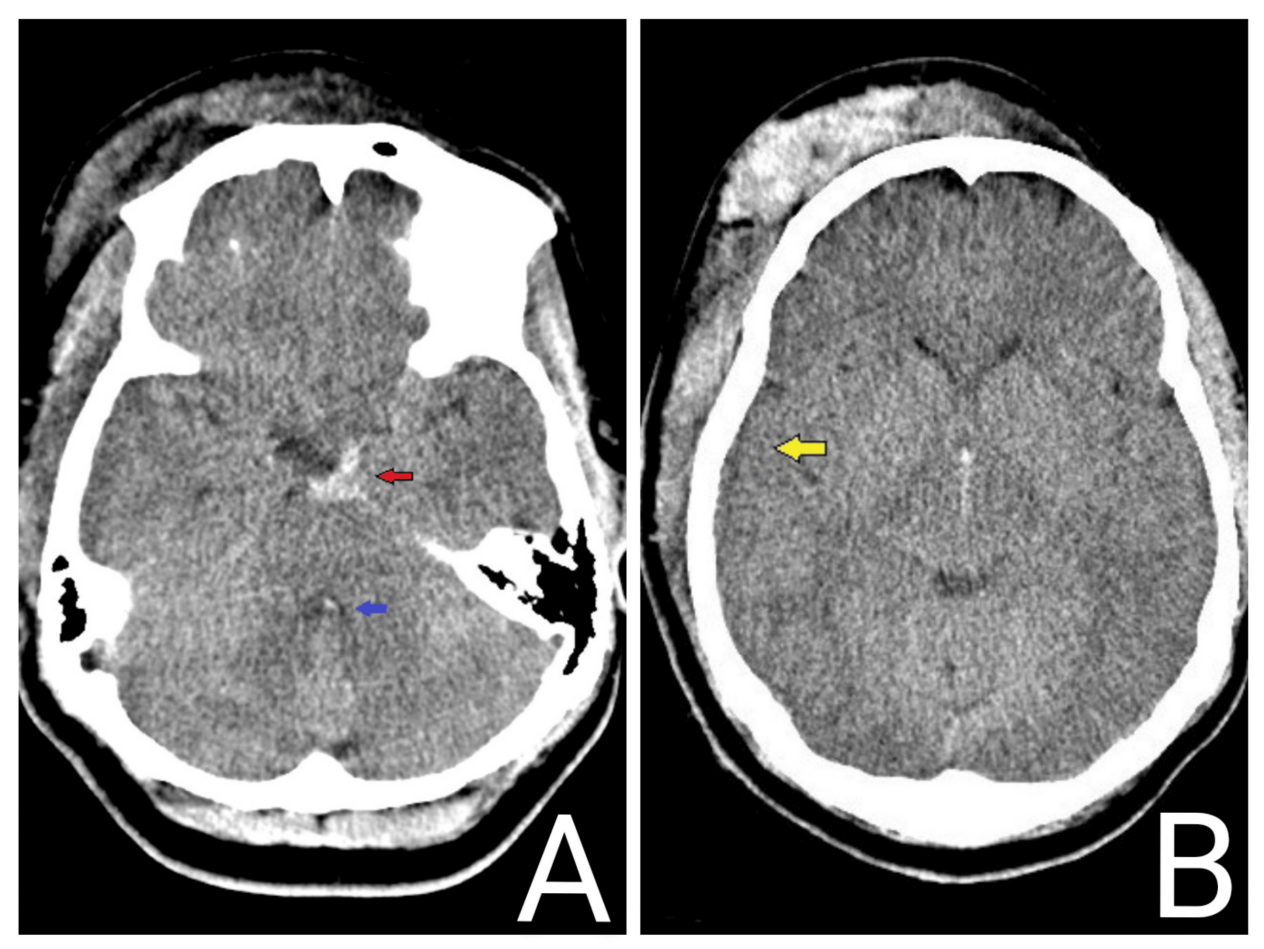
CT head of patient 1 Axial non-contrast CT of the head. (A) Subarachnoid hemorrhage (red arrow) and effacement of the fourth ventricle (blue arrow). (B) Sulci effacement (yellow arrow) and diffuse cerebral edema. On neurological examination, the patient presented with eyes closed to pain, not following commands, pupils 3 mm and minimally reactive bilaterally, localization in bilateral upper extremities, and withdrawal in bilateral lower extremities. CT: computed tomography

A 25-year-old G3P1112 woman at 35 weeks of gestation with a history of chronic hypertension, HSV, and previous cesarean delivery presented to the emergency department as a code 1 trauma after a MVA. Initial GCS was reported as 6, and the head CT demonstrated diffuse subarachnoid hemorrhage with diffuse cerebral edema. Representative CT image findings are shown in Figure 1. Fetal monitoring revealed late decelerations that improved slightly with lateral positioning. Due to the severity of the brain injury and the potential risk of rapid deterioration, an emergency cesarean section was performed. The patient delivered a viable male infant weighing 2,849 g with Apgar scores of 2, 4, and 5 at 1, 5, and 10 minutes, respectively. Following the cesarean section, the patient was admitted to the NICU for emergency placement of an external ventricular drain (EVD) and a brain tissue partial pressure of oxygen probe (PbtO₂ probe) for further management.

After three days of monitoring and no signs of intracranial hypertension, the probes were removed. A tracheostomy was performed on hospital day 8, followed by percutaneous endoscopic gastrostomy (PEG) tube placement on hospital day 9 for long-term enteral nutrition. Her ICU course was complicated by ventilator-associated pneumonia, which was treated with targeted antibiotic therapy. After 31 days, she was transferred out of the NICU. On day 44, her tracheostomy was successfully decannulated, following progressive ventilator weaning. The patient’s neurological recovery was slow but showed gradual improvement, with a retrospectively estimated Glasgow Outcome Scale-Extended (GOSE) of 3. She was discharged to a personal care home on day 69.

Patient 2

**Figure 2:**
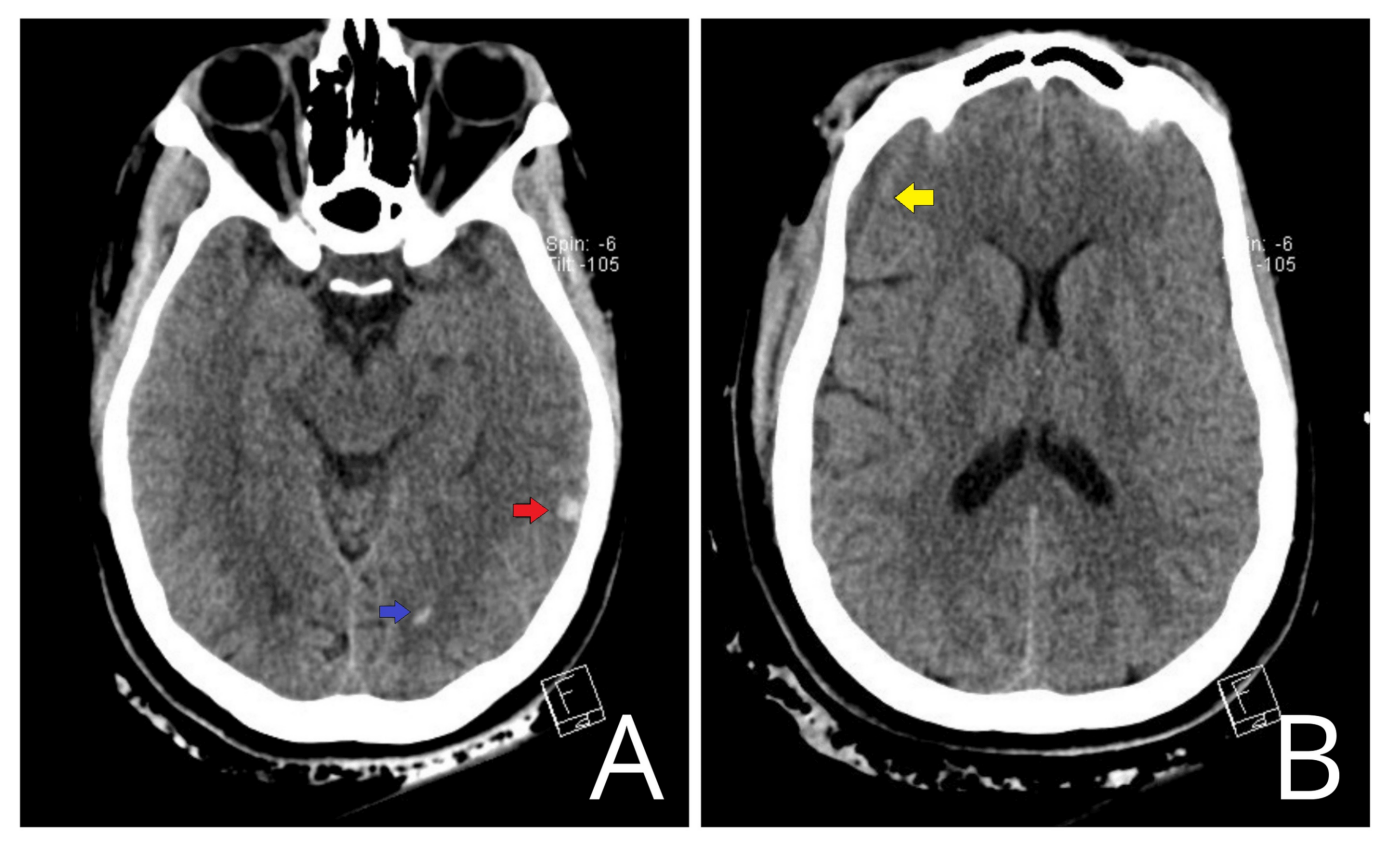
CT head of patient 2 Axial non-contrast CT of the head. (A) Temporal lobe contusion (red arrow) and intraventricular hemorrhage (blue arrow). (B) Trace subdural collection (yellow arrow). On neurological examination, the patient presented with eyes closed to pain, pupils 5 mm on the right and 4 mm on the left, both reactive to light, non-verbal, and extensor posturing in all extremities. CT: computed tomography

A 35-year-old G5P3013 woman at 20 weeks and 2 days of gestation presented to the emergency department as a code 1 trauma following an MVA. She was intubated on arrival for airway protection, and her GCS was reported to be 4. She received a blood transfusion for hemodynamic instability. Initial CT imaging revealed a right frontal bone fracture extending into the sphenoid and petrous apex, along with intracranial hemorrhage and cerebral edema. Relevant CT imaging findings are displayed in Figure 2. Since the fetus was determined to be previable due to gestational age, no acute obstetric intervention was performed. The neurosurgery team placed an EVD and a PbtO₂ probe. No treatment for intracranial hypertension was required, and the probes were removed on day 5.

On hospital day 7, she underwent percutaneous tracheostomy for ongoing respiratory failure and anticipated prolonged ventilator dependence. A PEG tube was also placed. She was transferred out of the ICU on hospital day 17 and was able to intermittently follow simple commands. Her hospital course was complicated by sepsis and acute colonic pseudo-obstruction, which were resolved after empiric antibiotic therapy and neostigmine, respectively.

Her pregnancy progressed without fetal compromise. She received a course of betamethasone for fetal lung maturation. Throughout the hospitalization, she continued to receive physical and occupational therapy. On day 120, she entered spontaneous labor and underwent an urgent but uncomplicated repeat cesarean section. A viable breech male infant was delivered with Apgar scores of 1, 6, and 8 at 1, 5, and 10 minutes, respectively. She was discharged home on day 125 in stable condition. The retrospectively estimated GOSE was 3. She required outpatient neurorehabilitation and standard postpartum follow-up.

Patient 3

**Figure 3:**
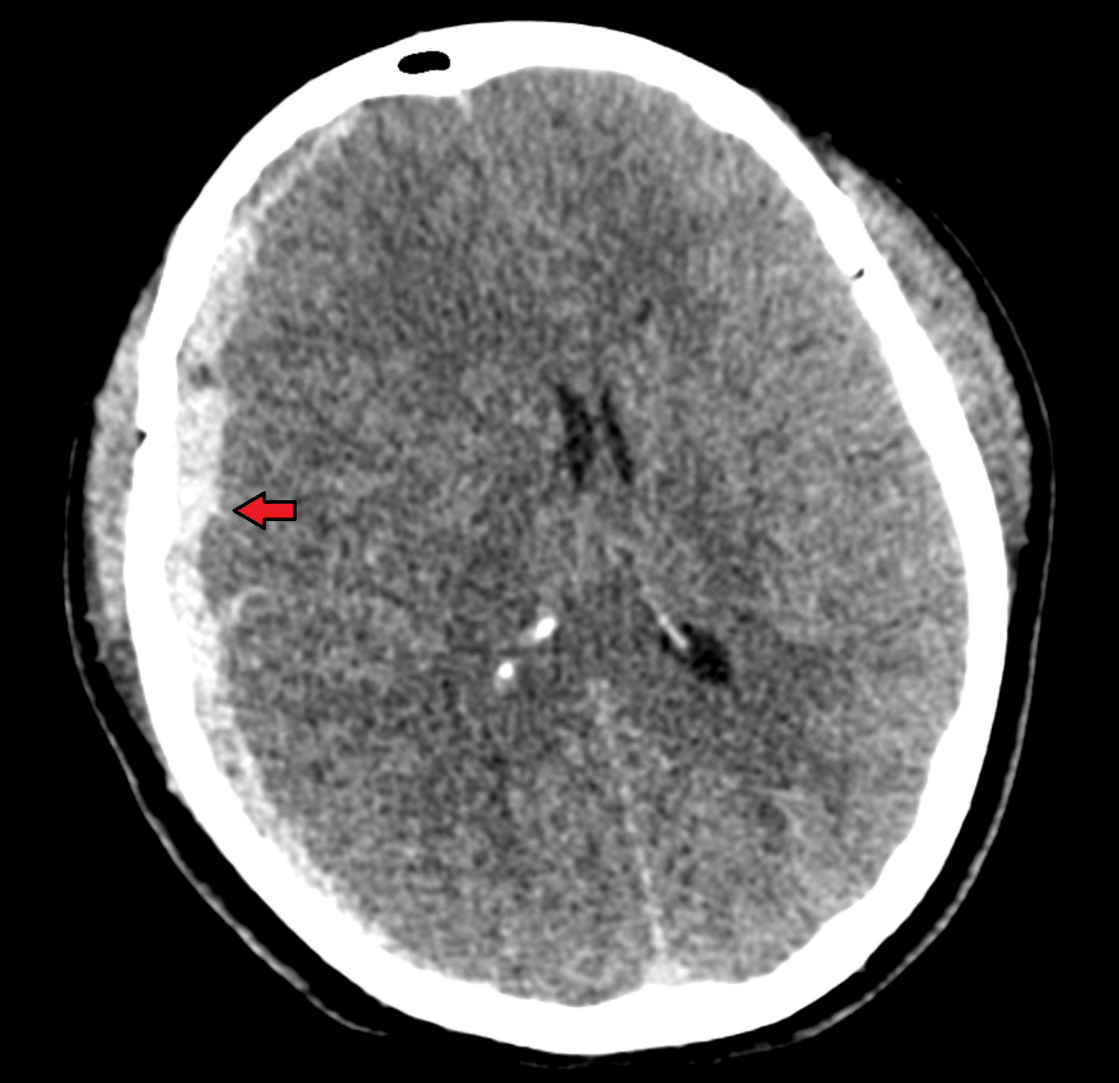
CT head of patient 3 Axial non-contrast CT demonstrating a large right subdural hematoma (red arrow) with significant mass effect and right-to-left midline shift. On neurological examination, the patient presented with eyes closed to pain, pupils 8 mm and non-reactive bilaterally, absent corneal reflexes bilaterally, non-verbal, extending the right upper extremity, no movement of the left upper extremity, and triple flexion of the bilateral lower extremities. CT: computed tomography

A 36-year-old G2P2 woman was admitted to the emergency department after being involved in an automobile versus pedestrian accident. She arrived as a code 1 trauma in ventricular fibrillation and underwent 15 minutes of cardiopulmonary resuscitation, after which return of spontaneous circulation was achieved. Upon initial assessment, she was found to have a GCS of 4, fixed and dilated pupils, and absent brainstem reflexes. Emergent bilateral chest tubes were placed for traumatic pneumothorax. The focused assessment with sonography for trauma exam revealed pregnancy, and a subsequent obstetric ultrasound dated the fetus at 14 weeks and five days. Since the pregnancy was found to be nonviable and the patient was in critical condition, no obstetric intervention was performed. A head CT revealed a 7-mm right-sided acute subdural hematoma with a 1.2-cm midline shift, diffuse subarachnoid hemorrhage, and a right frontoparietal skull fracture. Representative imaging is shown in Figure 3. She was immediately taken to the operating room for a right decompressive craniectomy for hematoma evacuation.

The patient was transferred to the NICU postoperatively, was on multiple vasopressors, and was in critical condition. Later that night, an abdominal CT raised concern for intra-abdominal injury, and an exploratory laparotomy was performed. Intraoperative findings included a grade 1 liver laceration, small hemoperitoneum, and an avulsed cecum from its lateral attachments, though bowel integrity remained preserved. Despite aggressive medical and surgical management, her neurological exam continued to worsen, and brain death was ultimately confirmed on day 3, which corresponds to a GOSE score of 1.

Patient 4

A 20-year-old G1P0 woman at 16 weeks and 1 day of gestation presented to the emergency department as a code 1 trauma after sustaining a gunshot wound to the head. On arrival, GCS was reported as 15 with no neurologic deficits.

**Figure 4:**
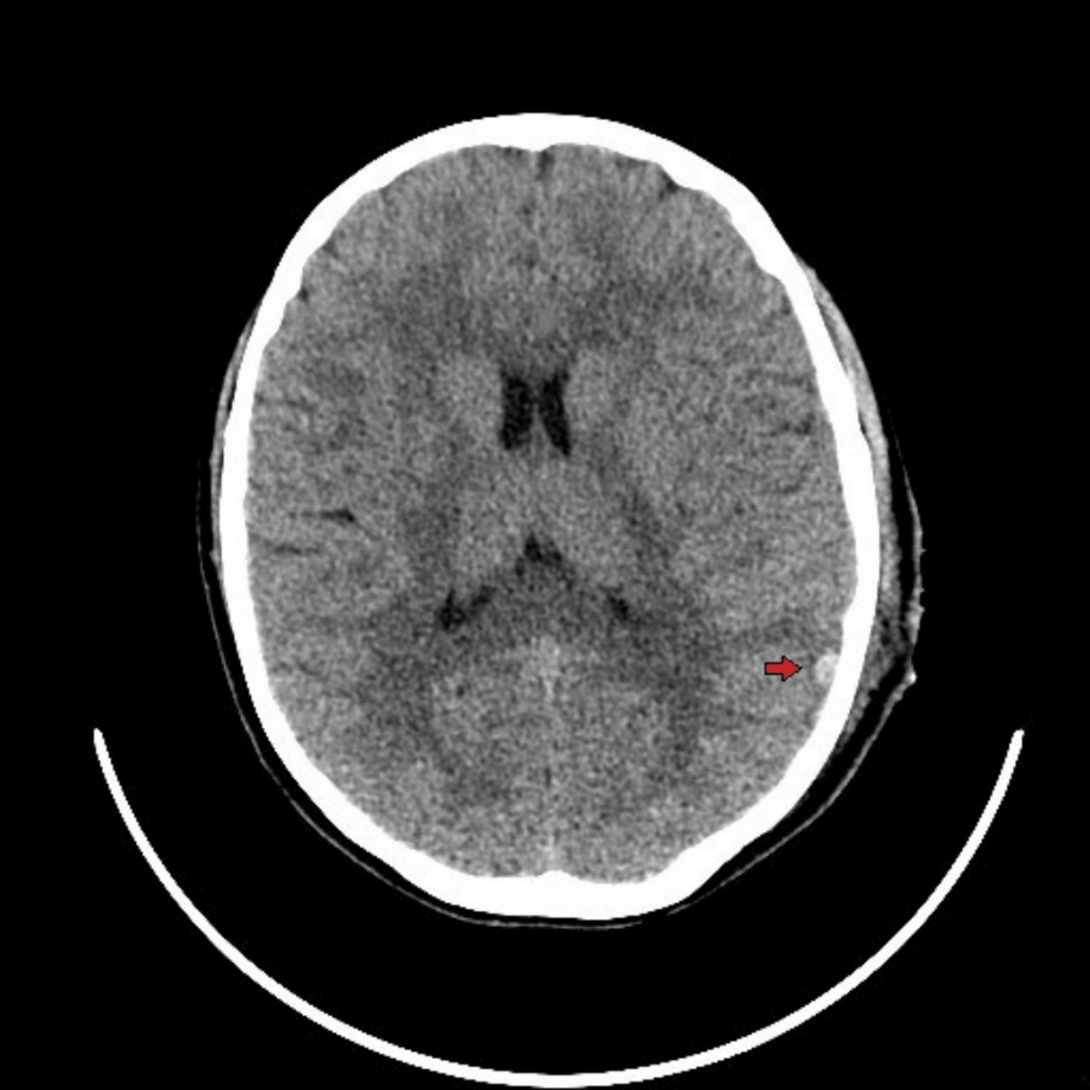
CT head of patient 4 Axial non-contrast CT of the head showing a small left parietal contusion (red arrow). On neurological examination, the patient presented as awake, alert, and oriented to person, place, and time, with fluent speech, pupils 3 mm in diameter and bilaterally reactive, and motor function with full strength in all extremities. CT: computed tomography

Head CT revealed a small left parietal contusion without underlying fracture, as shown in Figure 4. She was admitted to the NICU for observation. Obstetric evaluation confirmed a stable intrauterine pregnancy at 16 weeks and one day. The team discussed imaging safety and general pregnancy considerations and recommended follow-up brain imaging in the third trimester. A repeat head CT scan showed similar findings, with no change in the neurological exam. She was discharged home the next day in good condition, with routine obstetric follow-up. The retrospectively estimated GOSE was 8.

## Discussion

Over the last 10 years, we found four pregnant patients who suffered from TBI, resulting in 0.2% of all TBI cases admitted in our NICU. Although the incidence is not known precisely, it has been estimated that about 0.3% of pregnant women who require hospitalization are due to trauma [3]. Al Fauzi et al. conducted a systematic review of TBI epidemiology, management, and outcome in obstetric patients; they included 22 articles published between 1990 and 2020, and they found that the most common cause of injury was MVA [3]. In the United States, the annual crash rate for pregnant women has been estimated as at least 13 per 1,000 person-years, as compared to 26 crashes per 1,000 person-years among non-pregnant women; this highlights an important difference in the epidemiology of the studied condition [3]. Similarly, in our study over the last 10 years, we found four pregnant females suffered from TBI, resulting in 0.2% of all our TBI cases, and the most common mechanism of injury was MVA. Three of the four patients suffered severe injuries. This depicted heterogeneity in neurological presentations, timing of delivery, and discharge dispositions, with management demanding a multidisciplinary approach [5,11].

Our series aligns with various aspects of both studies, which involve large datasets and expert opinions. Picetti et al. conducted an international survey endorsed by the World Society of Emergency Surgery, which gathered responses from 122 physicians across 35 countries regarding the management of severe TBI during pregnancy [4]. They found out that significant variability among institutions was evidenced when managing TBI in pregnancy, particularly when it comes to sedation, ICP monitoring, and timing of delivery [4]. Our institution’s practices align with the most reported protocols, including early neurosurgical intervention, the use of ICP monitoring, and close collaboration with obstetrics and trauma surgery. Similarly, the EAST and SOGC guidelines recommend expediting delivery in critically ill patients with an imminent risk of deterioration at gestational ages of ≥24 weeks and ≥23 weeks, respectively [6,10]. For instance, due to the potential risk of death in case 1, it was determined to proceed with emergent cesarean delivery to avoid the need for a perimortem procedure. In contrast, in cases 2 and 3, no acute interventions from the obstetrics service were performed.

Regarding imaging modalities, most authors agree that radiation concerns should not compromise diagnostic accuracy, as doses <5 rad are not associated with a significant risk for fetal anomalies or pregnancy loss. EAST and SOGC recommend a CT scan when necessary. At the same time, the use of MRI can be considered in the appropriate clinical scenario, but it should not be based solely on the aim of reducing radiation exposure [6,10]. However, these guidelines do not specifically refer to TBI but instead offer recommendations for any trauma in pregnant patients. Darlan et al. proposed an algorithm specific to TBI, based on their local hospital procedures, recommending a head CT scan as the first-line evaluation [5]. Accordingly, all the presented cases underwent a head CT scan as part of their initial evaluation. Case 4, who was diagnosed with a mild TBI, had a repeat scan before being cleared for discharge. No patient required a brain MRI scan.

The most recent Best Practices in the Management of Traumatic Brain Injury guidelines, released in 2024 by the American College of Surgeons, provide insights about intracranial pressure (ICP) management, have some special considerations for special populations such as pediatric and older patients, but do not have a special section or recommendations on pregnant patients [9]. Similarly, the Brain Trauma Foundation guidelines remain central to evidence-based practice in neurotrauma care; nonetheless, they have not covered sections for the management of TBI during pregnancy [12].

Pregnancy, as well as polytrauma, has an increased risk of venous thromboembolism (VTE) [1,9]. Current TBI guidelines recommend considering VTE prophylaxis within 24 hours after injury in patients with low-risk nonoperative TBI, within 24 to 48 hours after injury in patients with moderate- or high-risk nonoperative TBI, and within 24 to 48 hours after surgery on those who underwent craniotomy or craniectomy with stable follow-up imaging [9]. The preferred agent for TBI patients is low-molecular-weight heparin (LMWH), including those with ICP monitors [9]. We used LMWH for VTE in our patients since it has an acceptable safety profile in pregnant patients [13].

TBI is a well-recognized cause of seizures and epilepsy, especially in pregnant women, where a modification of seizure propensity can be observed physiologically [1]. Currently, there is no consensus about seizure prophylaxis in TBI in pregnancy; nonetheless, the current recommendations for the general population contemplate antiseizure medication during the first week after TBI in high-risk patients to reduce early post-traumatic seizures [4,9]. The incidence of early post-traumatic seizures is correlated with the severity of TBI and other factors such as depressed skull fractures, SDH, ICH, GCS <10, or cortical contusions [9]. Levetiracetam remains the safest option in pregnant patients; however, early neurology consultation is recommended to mitigate fetal exposure to potentially other harmful antiseizure medications [13]. In our medical center, levetiracetam was the drug of choice in all four patients.

Following maternal stabilization, the EAST guidelines recommend continuous fetal monitoring for gestations >20 weeks, whereas the SOGC suggests monitoring at ≥23 weeks [6,10]. This is based on the rationale that helps to detect uteroplacental compromise and trauma-related complications, since fetal heart rate abnormalities can be the earliest sign of maternal hypovolemia. Patient one underwent fetal monitoring, but the decision to perform an emergency cesarean section was based on the maternal severity of injuries and risk of imminent deterioration. Previous gestations underwent a Doppler fetal heart rate assessment, with care escalated based on the clinical course. For patients who required non-obstetric procedures, such as the neurosurgical interventions described above, monitoring was performed before and after the procedure.

Long-term complications are another relatively unexplored area for this condition [2,14]. In a large Finnish population-based cohort, women with a history of TBI were found to have a modestly increased risk of adverse perinatal outcomes in subsequent pregnancies. Compared to controls, these patients had higher rates of preterm delivery, cesarean delivery, and neonatal intensive care unit admission [14]. In our series, long-term maternal and neonatal outcomes could not be assessed since the discharged patients did not return to our clinic for follow-up, and the newborn's medical records are not accessible.

We recognize the limitations of this study. Four patients over the course of 10 years is a small sample size that cannot lead to generalized conclusions. However, this illustrates the rarity of the condition, as our Level I Trauma Center is located in a densely populated area and has fully integrated neurosurgery and obstetrics services. Additionally, since none of the patients had follow-up visits in the clinic, no long-term neurological outcomes are available. The GOSE scores at discharge were calculated retrospectively from progress and rehabilitation consult notes, rather than from the comprehensive interview model; therefore, they should be interpreted with caution [15].

Nevertheless, this series supports several key points. From a neurocritical care perspective, ICP and PbtO₂ monitoring, along with timely interventions, can be effectively adapted for obstetric patients. Secondly, decision-making ultimately must be guided not only by gestational age but also by the evolving maternal neurologic condition [6]. Lastly, there is little data about fetal mortality. Still, it is estimated that the rate of fetal mortality after maternal blunt trauma is 3.4% to 38.0%, mainly from placental abruption, maternal shock, and maternal death [3]. Literature reports that perinatal outcomes can be favorable even in the context of severe TBI, as adequate fetal monitoring is conducted, delivery is performed in a timely manner, and maternal status is prioritized [10,14]. In our experience, the two viable infants were delivered without complications, and one patient was discharged with indications for routine obstetric follow-up. However, it is essential to note that due to the small sample size, these observations are not generalizable. Therefore, we emphasize the need for larger cohorts and pooled data from other level I trauma centers to enable more systematic reviews or meta-analyses. This is also an opportunity for medical societies across specialties to convene working groups to develop guidelines focused on this patient population.

## Conclusions

Our data confirmed the rarity of TBI in obstetric patients. Even though this represents an infrequent clinical scenario, the complications and treatment can be challenging for clinicians. Our 10-year experience illustrates the variability of presentations and outcomes. What emerges from these case presentations is not a universal approach, but rather an emphasis on coordinated efforts and multidisciplinary decision-making. Current trauma guidelines offer recommendations for obstetric patients, but not specifically for TBI. Pooled data from other level I trauma centers should be studied to inform the formulation of appropriate therapeutic directions, and the inclusion of this population in medical societies’ guidelines is necessary.
